# Deceptive behaviour in autism: A scoping review

**DOI:** 10.1177/13623613211057974

**Published:** 2021-11-26

**Authors:** Ralph Bagnall, Ailsa Russell, Mark Brosnan, Katie Maras

**Affiliations:** University of Bath, UK

**Keywords:** autism, deception, executive function, scoping review, social learning, theory of mind

## Abstract

The ability to deceive is a key milestone in social cognitive development for typically developing individuals. In this scoping review, we systematically searched the literature to summarise research on deceptive behaviour in autism and identify gaps in knowledge. Across the 28 studies identified, three main themes were synthesised, with seven subthemes: (1) *Deception ability and prevalence* (1a) gameplay deception; (1b) naturalistic deception; (2) *Psychological correlates of deception* (2a) verbal, intellectual and social ability; (2b) theory of mind (ToM) behaviours; (2c) executive function; and (3) *Social learning* (3a) training; (3b) social contexts. The findings challenge common stereotypes, showing that autistic individuals can and do engage in deception. However, many do so less frequently and less adeptly than typically developing individuals. Emerging evidence also suggests that autistic individuals (without co-occurring intellectual disability) may use compensatory strategies when engaging in deception, and that more skilled deceptive behaviour may arise later in life. Implications and directions for future research are discussed.

**Lay abstract:**

The ability to deceive others is an important skill that usually develops in early childhood. In this review, we give an overview of studies that have examined deceptive behaviour in autistic children, adolescents and adults. We separated the study findings into three main categories and seven sub-categories: (1) *Deception ability and prevalence* (1a) gameplay deception; (1b) naturalistic deception; (2) *Psychological processes in deception* (2a) verbal, intellectual and social ability; (2b) ability to understand others’ thoughts and beliefs; (2c) cognitive ability; and (3) *Social learning* (3a) training; (3b) social contexts. Contrary to some stereotypes, we found that autistic people can and do deceive but often find this more difficult than non-autistic people. We also found that autistic people may use different psychological processes than non-autistic people when deceiving and may get better at deception in adulthood.

The ability to deceive others reflects typical social cognitive development: drawing upon social understanding, learning and cognition (Hala & Russell, 2001; Lee, 2013). Controlling the degree to which one is truthful is essential for navigating a range of social contexts (Spence et al., 2004; Vrij, 2008). Judging which information to include, omit or reconstruct contributes to impression management, the initiation and maintenance of relationships and protection against social threats (Bowker & Tuffin, 2006; Choshen-Hillel et al., 2020; Levine & Schweitzer, 2015; Maras et al., 2021; Metts & Grohskopf, 2003). However, it has been suggested that individuals with autism spectrum conditions (henceforth, autism) rarely, if ever, intentionally deceive others (e.g. Baron-Cohen, 2007). Thus, understanding the ability of autistic individuals to engage in deception, and which social cognitive factors underpin this, can provide insight into a broad range of social behaviour.

At a theoretical level, there are several areas that indicate that deception will prove challenging to autistic people. Theory of Mind (ToM; Baron-Cohen et al., 1985; Premack & Woodruff, 1978) – the capacity to understand and predict others’ mental states – is thought to be a necessary prerequisite to deception (Walczyk & Fargerson, 2019). ToM is positively associated with understanding, producing and maintaining deception in typically developing (TD) young people between the ages of 2 and 19 years (Sai et al., 2021; Sarah Lee & Imuta, 2021). However, while not universal, difficulties in understanding others’ mental states are common for autistic people in experimental tasks (Brewer et al., 2017; Peñuelas-Calvo et al., 2019; Yirmiya et al., 1998) and naturalistic settings (Livingston & Happé, 2017). As such, a diminished ability to recognise and understand others’ mental states (e.g. emotions and beliefs) may increase the difficulty of deceptive behaviour for autistic people.

Deception is often more cognitively demanding than being truthful, with liars experiencing greater difficulty and response delay than truth-tellers (Caso et al., 2005; Suchotzki et al., 2017). Executive function, referring to a range of psychological processes involved in goal-orientated cognition (Zelazo & Müller, 2011), positively (though modestly) relates to understanding, producing and maintaining deception (Sai et al., 2021). For example, working memory underpins the maintenance of retrieved episodic information during lying in TD individuals (Debey et al., 2014; Maldonado et al., 2018; Sporer, 2016). However, difficulties in executive functioning (Demetriou et al., 2018), including domains of working, short- and long-term and episodic memory (Cooper & Simons, 2019; Crane & Maras, 2018; Desaunay et al., 2020; Habib et al., 2019) are common in autism throughout the lifespan. Thus, difficulty with selecting, retrieving and maintaining lie-relevant information may further increase the cognitive demand of deception for autistic people.

Furthermore, interpersonal deception is inherently social. Buller and Burgoon (1996) emphasise that interpersonal deception is embedded in the interaction between liar and recipient, each of whom draw upon social information (such as social cues, norms and contexts) to make sense of their encounter. For example, TD children are socialised to learn the utility of lying and model their deceptive behaviour from observing adults (Engarhos et al., 2020; Lavoie et al., 2015; Talwar & Crossman, 2011). However, autistic children and adults can have difficulty attending to social stimuli (Chawarska et al., 2012; Chevallier et al., 2015; Hedger & Chakrabarti, 2021) and may therefore have fewer opportunities to learn from social experience (Bushwick, 2001; Chevallier et al., 2012a; Ma et al., 2019; Vivanti et al., 2016). For example, autistic adults appear to be poorer at detecting deception than TD comparison individuals, possibly due to decreased social engagement in situations from which they could learn deception-relevant behavioural cues (Williams et al., 2018). Indeed, TD adults’ ability to successfully detect deception is positively associated with their ability to deceive others, and this ‘deception-general’ ability may be developed and refined through social learning (Wright et al., 2013).

There are currently two existing reviews on the topic of autism and deception.^1^ An early review (Sodian & Frith, 1993) detailed three studies of deception in autism (Baron-Cohen, 1992; Russell et al., 1991; Sodian & Frith, 1992), proposing a marked lack of ability to deceive. Sodian and Frith (1993) tentatively suggested that autistic children’s difficulty with deception is primarily due to a diminished ability to understand and manipulate (false) beliefs. A second, later review (Kisamore et al., 2018) discussed two studies of deception in autism (Bergstrom et al., 2016; Ranick et al., 2013) in relation to behavioural skills training. Kisamore et al. (2018) suggested that interventions using social rule-learning and behavioural reinforcement could help autistic children and adolescents learn to tell pro-social lies while presenting appropriate behavioural cues.

In light of these mixed findings, a cohesive synthesis of the literature was undertaken. The objective of this scoping review was to collate the breadth of research to date about autism and deceptive behaviour, describe the evidence base, highlight key theory and concepts, and identify current gaps in knowledge.

## Method

A scoping review methodology was chosen to enable the mapping of evidence, concepts, and theory of research on this topic (Tricco et al., 2018). Scoping reviews allow for a broader overview of a given topic than other forms of knowledge synthesis (e.g. systematic reviews) and aim to collate and describe an evidence base (Munn et al., 2018). Given the lack of a recent overview of research on deceptive behaviour in autism, a scoping review methodology was chosen to establish current understanding as well as gaps in knowledge.

It is noted that previous research has incorporated measures of deception understanding as part of a battery of ToM tests (e.g. the Strange Stories task: Happé, 1994) and a smaller number have examined autistic individuals’ ability to detect deception in others (e.g. Williams et al., 2018). The present scoping review was initially registered as an examination of research relevant to all aspects of deception and autism, including understanding of deception and its detection. Due to the vast number of studies that have included a measure of deception understanding (often, as stated, as part of a battery of ToM tests), this focus was subsequently narrowed to studies which report on autistic individuals’ deceptive behaviour. This decision was made to allow for a more detailed summary of the available literature on actively deceptive behaviour.

The current study followed the five-stage scoping review framework described by Arksey and O’Malley (2005) (a) identifying the research question; (b) identifying relevant studies; (c) study selection; (d) extracting (or ‘charting’) the data; and (e) collating, summarising, and reporting the results. In line with guidance from the Preferred Reporting Items for Systematic Reviews and Meta-Analyses Extension for Scoping Reviews (PRISMA-ScR; Tricco et al., 2018), a scoping review protocol was developed and pre-registered with the Open Science Framework.

### Eligibility criteria

The review used the following inclusion criteria: (1) Only sources relevant to the subjects of autism and deceptive behaviour were included (e.g. studies which exclusively tested deception detection or understanding were ineligible); (2) Due to time and budgetary restraints, only sources in English were included; (3) Quantitative, qualitative and mixed-methods research designs, as well as reviews and theoretical papers, were all eligible for inclusion if they contained relevant material not covered in other published work; (4) As per guidance for Joanna Briggs Institute (JBI) reviews (Peters et al., 2020), unpublished (‘grey’) literature was eligible for inclusion. No limitations were placed on publication date.

### Search strategy

Searches were conducted in Scopus, PubMed, and PsycInfo databases. An initial search of PubMed and PSYCHInfo using controlled vocabulary terms was conducted ( ‘Autism Spectrum Disorders’ AND ‘Deception’) across all fields. This produced 129 results (PubMed = 42; PSYCHInfo = 87). As per Peters et al. (2020), titles, abstracts, keywords and index terms were analysed and led to the inclusion of further search terms. *Autism Spectrum Disorders* was truncated to ‘autis*’ and broadened to include ‘ASD’ and ‘asperger*’. *Deception* was broadened to include ‘deceive*’, ‘deceptive’ and ‘lying’.

The final search strategies used ‘OR’ and ‘AND’ Boolean operators to combine controlled vocabulary terms with free-text terms. Appendix A presents the full, final search strategies for the three databases. Alerts for new articles were set on Scopus and PsycINFO (this option was unavailable on PubMed at the time). The final search was conducted on 03 November 2020. Articles were imported into the reference management software EndNote™ X9 (Endnote, 2013). Reference lists of all articles selected for inclusion were screened for further sources.

### Study selection and data extraction

A screening tool containing the inclusion criteria was used during the screening process. The first author used the screening tool to assess whether studies obtained through database searches were eligible for inclusion on the basis of title and abstract information. Studies which met eligibility were then screened for inclusion by the first author on the basis of their full text. A randomly selected 20% of studies at the title and abstract stage and 50% of studies at the full-text stage were screened independently by the first author and a research assistant, with 84% and 85% agreement, respectively. Disagreements were resolved through discussion.

**Figure 1.**
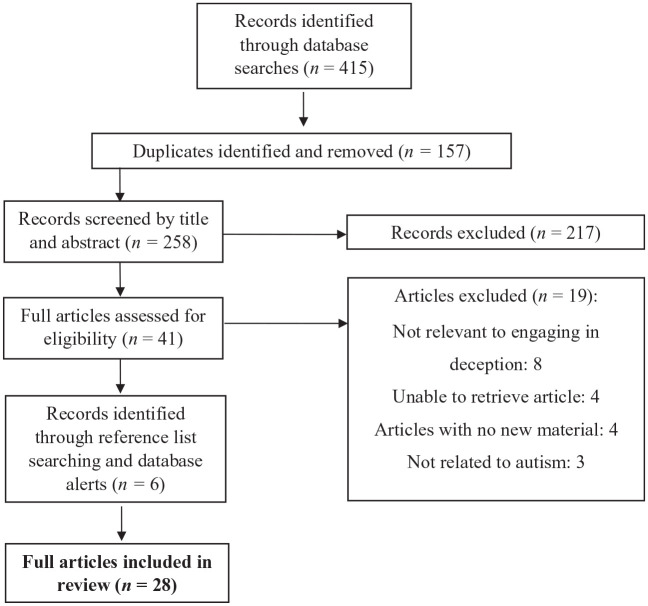
PRISMA flow diagram detailing study selection process.

Using a JBI template (Peters et al., 2020), a data extraction form was developed to capture relevant study characteristics. The form captured the following characteristics: (1) Title; (2) Author(s); (3) Year of publication; (4) Country of origin; (5) Participant sample; (6) Design / Methods; (7) Aims; (8) Findings. The first author extracted the data from studies assessed as eligible for inclusion in the review. To ensure that the data extraction form was applied consistently, 20% of the final extractions were verified and approved by the fourth author. Figure 1 presents a flow diagram of the study selection process.

### Summarising the data

Study characteristics were reviewed by the first author to identify major themes. Through discussion with the other authors, the main areas of focus within the data were determined to relate to three key themes: (1) Deception ability and prevalence; (2) Psychological correlates of deception and (3) Social learning. Further review and discussion of the study characteristics indicated clusters of sub-themes, which more precisely captured key theory and concepts across the data. Ultimately, three main themes and seven sub-themes were produced by the first author in collaboration with the other authors. Numerical analysis of study characteristics (i.e. year of publication; country of origin; theme) was performed. The themes and related findings are presented in the ‘Results’ section.

### Community involvement

Members of the autism community were not involved in the design, implementation or interpretation of this study.

## Results

The 28 studies screened as eligible for inclusion are presented in Table 1. All studies were published between 1989 and 2021. Of the 13 studies published between 2011 and 2021, five were published between 2019 and 2021. The majority of studies were quantitative (*n =* 26), employing case-control designs with participants with an autism spectrum condition. Only two studies used qualitative methods. Studies were conducted internationally, with the United Kingdom producing the most research with eight published studies (29%), followed by five studies from China (18%) and four studies from Canada (14%).

**Table 1.** Study codes and citations.

Code	Citation
01	Barbaro & Dissanayake (2007)
02	Baron-Cohen (1992)
03	Bergstrom et al. (2016)
04	Davidson & Henderson (2010)
05	Fisher & Happé (2005)
06	Gowen et al. (2008)
07	Hughes & Russell (1993)
08	Hughes et al. (1997)
09	Jaarsma et al. (2012)
10	Li et al. (2011)
11	Lu et al. (2019)
12	Ma et al. (2019)
13	Oswald & Ollendick (1989)
14	Pilowsky et al. (2000)
15	Reinecke et al. (1997)
16	Russell et al. (2003)
17	Russell et al. (1991)
18	San José Cáceres et al. (2014)
19	Sodian & Frith (1992)
20	Talwar et al. (2012)
21	van Tiel et al. (2021)
22	Yang et al. (2017)
23	Yi et al. (2014)
24	Yirmiya, Solomonica-Levi, & Shulman (1996)
25	Yirmiya, Solomonica-Levi, Shulman, & Pilowsky (1996)
26	Yokota & Tanaka (2013)
27	Yokota & Tanaka (2020)
28	Zhang et al. (2019)

Citations are noted by * and code number in the Reference list.

The total number of participants from studies in this review (where these data are available) is 1469, 641 (44%) of whom had an autism diagnosis or met diagnostic criteria for an autism spectrum condition. The studies mostly involved autistic male majority samples (*n* = 21), with gender not reported in six studies; 72% (*n =* 464) of autistic participants were male. The mean age of autistic participants was 12.86 years (*SD* = 7.32 years), ranging between ages 5 to 34 years. Twelve of the studies were with exclusively child participants (43%), two were with adolescents (7%), and four were with adults (14%). Five studies had mixed samples of children and adolescents (18%), four had mixed samples of children, adolescents, and adults (14%) and one had a mixed sample of adolescents and adults (4%).

**Figure 2.**
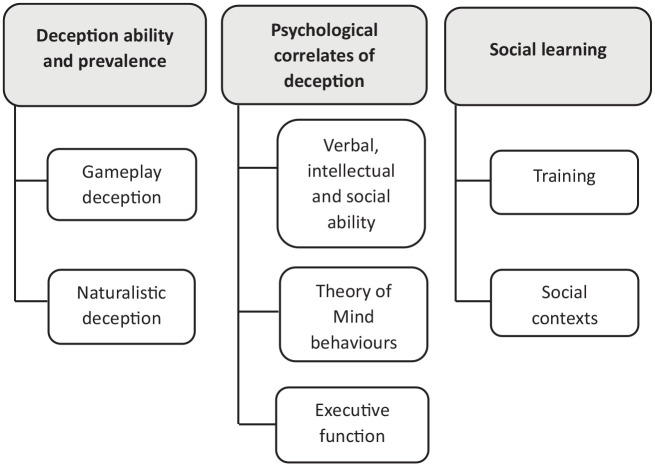
Thematic map of data synthesis.

Figure 2 presents the three overarching themes and seven subthemes identified across the studies. In the Supplementary Materials, details of all the 28 studies are included in Appendix B; an overview of study characteristics in Appendix C; study themes and subthemes in Appendix D; and a deception terminology index in Appendix E.

### Theme 1: deception ability and prevalence

#### Gameplay deception

Twenty two studies examined deceptive behaviour through performance on gameplay paradigms, in which verbal or non-verbal deceit is the instructed aim of the task. Ten studies (11, 12, 14, 22, 23, 24, 25, 26, 27, 28) used a hide and seek paradigm. These tasks require participants to (1) select a location (usually one of three boxes) in which to hide an object and then (2) deceptively point to a location so the opponent fails to find the object. The opponent will always choose the location provided by the participant, and the participant wins the trial if they successfully point to the empty location. Four studies (11, 22, 23, 28) showed that autistic children were significantly less likely than TD children to successfully pass the hide and seek task through deceiving an opponent across five successive trials (out of a maximum of 10 trials).

Three studies (14, 24, 25) used a variation of the hide and seek task. These studies tested two aspects of gameplay deception – (1) manipulating behaviour (i.e. hiding an object and laying or erasing false trails to deceptively lure an opponent to a false location) and (2) understanding manipulation of beliefs (i.e. answering a prediction question). In one study (24), only 43% of the 14 autistic adolescents and adults laid false trails or erased all trails and lied about the location of the hidden object (see *Theory of mind behaviours* section of the review for the false belief prediction findings). The autistic group children were significantly less likely to deceive than TD children, of whom 94% of 16 successfully passed the task. However, the autistic group children were not significantly different from adolescents and adults with intellectual disability (ID), of whom 67% of 15 passed the task (24). A second study (25) similarly reported that TD children were significantly more likely to successfully deceive than autistic children, adolescents and adults, though no significant differences were found between the autistic group and adults with ID. A third study (14) reported that a group comprised of children and adolescents with schizophrenia were significantly more likely to pass the deception task than the group comprised of autistic children, adolescents and adults, However, there were no significant differences in deception task performance between the autistic group and TD children. Nor were there significant differences in deception performance between TD children and children and adolescents with schizophrenia (14).

Three studies used the windows task (07, 16, 17), a variation on the hide and seek paradigm in which the (pre-hidden) object is visible only to the participant through transparent windows. In two of these studies (07, 17), compared with 4-year old TD children and children with ID, autistic children, adolescents and adults were less likely to point to the empty box to deceive their opponent and were more likely to persistently point to the box containing the object across multiple trials. One study (16) found no difference in deception performance on the windows task between autistic and ID children (see *Executive function* section for further findings from this study).

Seven studies employed the penny hiding game (02, 05, 06, 08, 13, 15, 18), a non-verbal gameplay task in which participants hide a penny in one hand to prevent an opponent from guessing in which hand the penny is hidden. In three studies, autistic children (18) and adolescents (02, 13) failed significantly more frequently than comparison groups to hide the penny’s location from their opponent during the task (such as through leaving the empty hand open or failing to hide the hand-penny transfer). One study (06) reported slightly lower performance by autistic than TD adults, though did not report descriptive or inferential statistical results. (See *Training* section for studies 05 and 15).

Other studies (08, 21, 26, 27) have reported more successful gameplay deception by autistic participants. One study (08) found no difference in autistic and TD childrens’ performance on the penny hiding game. Another study (26) reported that autistic children were only marginally less successful than TD children at concealing information during a hide and seek paradigm, though performed equally well at misdirecting an opponent away from the location of a desired object. In a further study (27), autistic children were just as accurate as TD children in producing deceptive ‘yes’/’no’ responses to misdirect a computerised opponent from the location of a hidden object, though were significantly slower at doing so. A recent study (21) found that autistic adults were equally likely to successfully deceive a computerised opponent as TD adults, and on trials in which deception was optional though would lead to a higher score, autistic adults were more likely to engage in deception than the TD group.

#### Naturalistic deception

Seven studies examined naturalistic displays of deception in autism. These studies describe how autistic people spontaneously engage in deception outside of experimental competitive gameplay settings. Four studies (10, 12, 20, 26) used an experimental temptation resistance paradigm, in which children were left alone in a room and instructed to not to look at a forbidden item and later asked if they looked. As with the TD comparison group, many autistic children looked at the item and engaged in verbal self-protective deception to cover their transgression. In the autistic group, this ranged from 50% of 16 participants (12) to 93% of 15 participants (10). However, three studies (10, 12, 20) found that autistic children struggled to maintain their verbal self-protective deception when asked about the identity of the object as a follow-up question. Significantly fewer autistic children than TD children feigned ignorance of the identity of the item, ranging from 7% of 14 autistic participants (10) to 62.5% of eight participants (12). These findings indicate that while autistic children often engage in naturalistic verbal deception, many have reduced ability to maintain their deception during follow-up questioning (i.e. control ‘semantic leakage’). However, one study (26) found no difference between autistic and TD children’s ability to offer a plausible verbal deceptive response to follow up questioning. Furthermore, another study (10) reported that during an undesirable gift paradigm (in which participants are asked whether they like the undesirable gift) most autistic children engaged in verbal pro-social deception and were equally convincing as TD children (i.e. offering a simple response without elaboration).

Two qualitative studies analysed autobiographical narratives of autistic adults in relation to naturalistic deception. The earlier of the two studies (04) proposed that autistic adults describe using deception as a way of managing their identity, through choosing when not to disclose autism diagnoses or to disguise their autism. The authors suggest that deception can therefore be used by autistic people as a self-protective strategy to navigate social contexts which present the risk of stigma (04). In the second study (09), verbal deception is again described as a form of self-protection. However, the authors also include excerpts from autistic adults’ narratives which indicate a reluctance to deceive others, linked to physiological, psychological and moral aversion to dishonesty (09).

Two studies to date have examined naturalistic non-verbal deception in autism. One study (01) found that autistic children present less effective self-presentation display rules (e.g. failing to maintain a neutral facial expression) than TD children when engaging in deception. However, a second study (10) found that autistic children were equally as likely as TD children to use non-verbal behaviours (i.e. nodding) when giving deceptive responses to being asked if they liked an undesirable gift.

### Theme 2: psychological correlates of deception

#### Verbal, intellectual and social ability

Fourteen studies measured the relationship between verbal, mental or chronological age and deceptive behaviour. Higher verbal mental age (VMA) was a significant predictor of success on gameplay deception tasks in autistic children and adolescents with and without co-occurring ID (18, 25, 26). VMA was also found to significantly and negatively correlate (*r* = −0.73) with learning speed on a gameplay deception task in autistic children without co-occurring ID (23). Autistic childrens’ IQ was also significantly correlated with learning speed on this task (*r =* −0.76), though when age and VMA were controlled for this correlation reduced (*r* = −0.48) and was non-significant (23). Another study (27) reported that age positively predicted response speed in autistic and TD children during a computerised gameplay deception task, though did not predict accuracy.

While these studies indicate that increased verbal ability is related to deception in autism, others report a less clear relationship. One study (19) reported that the majority of autistic children and adolescents with VMA of between 4 and 5 years failed a gameplay deception task that was passed by the majority of non-autistic 4-year old children and children with ID with a VMA of 5 years. Of five autistic children and adolescents with VMA of between seven and 12 years, 60% were able to pass the task. However, when false-belief understanding was included in the analysis as a covariate, neither group membership (autistic, ID, TD) nor VMA were significant predictors of deception ability (19). Another study (10) found that propensity for naturalistic verbal self-protective deception was significantly and negatively related to VMA (*r =* −0.57) in autistic children, while a non-significant relationship was found for TD children (*r =* −0.09). The same study reported that VMA was not significantly related to semantic leakage control nor propensity or ability for naturalistic verbal pro-social deception in the autistic group, though was related to semantic leakage (*r =* 0.69) in TD children (10). Two studies (01, 14) found no relationship between gameplay or naturalistic deception with VMA or vocabulary in autistic children, adolescents and adults. One study (24) found no significant differences in the mental or chronological ages between autistic children, adolescents and adults who were and were not able to successfully deceive in a gameplay task.

Two studies reported that performance on gameplay deception tasks was significantly and positively related to social interaction skills in autistic and non-autistic children (18) and adolescents (13, 18). One study (22) found no relationship between social responsivity or social communication with gameplay deception in autistic children, though did not measure TD children.

#### Theory of mind behaviours

Fourteen studies examined the relationship between theory of mind (ToM) behaviours and deception in autism. Eight studies used measures of first-order false belief (ToM) understanding, including recognition of participants’ own false beliefs (02) and predicting the false beliefs of others (05, 10, 12, 17, 19, 20, 23) through tests such as the Smarties task (Gopnik & Astington, 1988). Two studies (17, 19) reported a positive association between first-order ToM and gameplay deception performance (both non-verbal and verbal) in TD and autistic children and adolescents. One study (12) found that frequency of autistic childrens’ attempts at gameplay deception correlated positively (*r =* 0.46) with first-order false belief understanding. One study (05) measured the impact of first-order ToM training on performance on a gameplay deception paradigm, with significant improvement shown among autistic children and adolescents who received training compared with those who received no training.

Three studies (14, 24, 25) examined first-order ToM within a gameplay deception paradigm through the addition of follow up questions asking participants to predict the effect of their deception. In one of these studies (24), 43% of 14 autistic adolescent and adult participants successfully passed the deception task, though, among those who passed, only 33% could correctly predict where the opponent would look for the object. This was significantly fewer than the 80% of 15 TD children who understood how their actions had manipulated the opponent’s beliefs (24). However, in another study (14), 83% of the autistic group (comprising 12 autistic children, adolescents and adults) passed the deception task, 70% of whom also correctly predicted where the opponent would look for the hidden object. There were no significant differences between the autistic group and the TD group (comprised of children). Each of these groups was significantly outperformed by children and adolescents with schizophrenia, of whom 100% of 12 participants were able to successfully pass both the deception task and the prediction question (14). In the third of these studies (25), where groups were matched on performance mental age, 8% of 12 autistic children, adolescents and adults passed both the deception task and prediction question across two trials, in comparison to 29% of 14 children, adolescents and adults with ID, and 79% of 14 TD children. The results were broadly similar when groups were matched on VMA (25).

One study (21) inferred autistic adults’ ToM ability based on performance during a gameplay deception paradigm, with the autistic group’s deception performance found to be equivalent and often superior to TD group performance (see *Social contexts* section of the review for further detail).

In other studies, the role of first-order ToM for deception ability and prevalence in autism is less clear cut. One study (20) examined naturalistic verbal deception (i.e. tendency to lie to disguise a transgression) in autistic and TD children and found that, when groups were collapsed into liars and truthtellers, children who deceived had higher first-order false belief scores than children who told the truth. However, there was no significant difference in first-order false belief scores between autistic and TD groups, or differences in first-order belief scores within autistic and TD groups between children who lied or told the truth (20). Other studies reported that they found no significant association between first-order belief understanding and naturalistic self-protective (10, 12) or pro-social verbal deception (10) or gameplay deception (23) in autistic children. Two studies (07, 16) inferred the role of first-order ToM understanding by comparing autistic children and adolescents’ gameplay deception performance in either social or non-social conditions, finding little evidence for ToM being a primary explanatory factor in deception task ability (see *Executive function* section of the review for further detail).

Only two studies (10, 20) tested second-order false beliefs through measures such as the Ice Cream task (Perner & Wimmer, 1985). In one of these studies (10), the ability to maintain naturalistic self-protective deception during follow-up questioning (i.e. semantic leakage control) was associated with second-order false belief understanding in TD though not autistic children. In the same study, second-order false belief understanding was also not associated with the prevalence of naturalistic self-protective or pro-social verbal deception in autistic children (10). In the second study (20), no significant effect of second-order false belief understanding on naturalistic deception was found in either TD or autistic children under an identical paradigm. However, the TD group had higher second-order false belief understanding than the autistic group and were also significantly more likely to lie (20).

#### Executive function

Eight studies examined the role of executive function in relation to deception in autism. Three studies (07, 16, 19) investigated the role of executive function through performance on deception and non-deception gameplay tasks. The first study (07) tested whether removing ToM demands in gameplay deception tasks would reveal that executive function processes (i.e. inhibiting attention from salient visual information) could explain differences in performance between autistic and comparison groups. The introduction of a ‘no opponent’ condition, therefore not requiring ToM, failed to improve gameplay deception task performance in autistic children, adolescents and adults. The researchers proposed that autistic participants’ difficulty with certain deception tasks may primarily be due to executive (dys)function (07).

A later study (16) further tested the ToM versus executive function hypothesis through a simplified, automated version of the same task across three conditions (no opponent, opponent non-deceptive, opponent-deceptive). Autistic children’s performance was consistent across the three conditions, therefore the study failed to find conclusive evidence for either a ToM or executive function explanation. The authors suggest that reduced task difficulty may have removed differences between deception and non-deception conditions (16). However, another study (19) further downplayed the role of executive function on deception task performance, reporting that autistic children’s performance was effective on non-deception tasks requiring them to sabotage an opponent’s access to a reward. On a parallel gameplay deception task requiring the manipulation of beliefs through verbally lying or deceptively pointing, autistic childrens’ performance was generally poorer than TD comparisons (19). The authors proposed that performance differences between sabotage (non-deception) and deception tasks could therefore not be explained by a lack of task understanding or reduced behavioural control, and that diminished ToM therefore primarily explained poor deception ability. Similar conclusions were reached in a gameplay deception study (24), in which participants needed to predict the outcome of their deceptive laying of false footprints. Autistic adolescent and adult participants had difficulty doing so despite the footprints providing a salient visual cue on which to base their prediction (24). From this, the authors suggested that ability to disengage attention from salient objects played a smaller role than ToM for explaining poor performance and understanding during gameplay deception tasks (24).

Three studies (12, 23, 27) tested deception performance in relation to specific measures of executive function domains. A recent study (12) reported that working memory, though not inhibitory control, positively correlated with propensity (*r =* 0.49) and frequency (*r =* 0.62) of gameplay deception in autistic children. Working memory also positively correlated with autistic childrens’ propensity (*r =* 0.49) and ability (*r =* 0.7) of self-protective naturalistic verbal deception at marginal significance (12). Another study (27) reported that greater capacity for planning predicted faster responses (though not accuracy) on a computerised gameplay deception paradigm among autistic children, while simultaneous processing, successive processing and attention were unrelated. In a further study (23), executive function measured by the Dimensional Change Card Sort task significantly correlated with strategic deception performance in autistic (but not TD) children, though this effect reduced in size (*r* = −0.48) and non-significance once age and VMA were controlled.

One study (05) measured autistic children and adolescents’ gameplay deception in relation to executive function training (see *Training* section of the review).

### Theme 3: social learning

#### Training

Three studies investigated the extent to which deception ability in autism can improve through training. An intervention training study (15) reported that 100% of the three young autistic people with co-occurring ID and limited verbal ability learned to present more effective deceptive responses during a non-verbal gameplay deception task. A behavioural skills training intervention study (03) reported that within a few sessions of rule teaching, role-play and feedback, 100% of the three autistic children learned to use socially appropriate verbal deception, matched with a sincere vocal tone and appropriate facial expression. This was generalised using an identical context (e.g. receiving an undesired gift) with adults not present during the training phase. One study (05) reported that autistic children and adolescents who received executive function and ToM training demonstrated superior performance on gameplay deception tasks than those who received no training.

#### Social contexts

Eight studies examined how the reading of social contexts impacts deceptive behaviour in autism. Here, the reading of social contexts is distinguished from ToM-specific abilities due to encompassing the effects of a broader range of social information and understanding. Three studies (11, 22, 23) suggest that diminished social understanding may act as a barrier for autistic childrens’ ability to learn when they are being deceived and to deceive in retaliation. For example, one study (22) reported that during a gameplay deception task, autistic children were slower to learn from social cues indicating they should distrust an opponent and engage in retaliatory deception. During trials with non-social cues, autistic and TD children learned to deceive at a comparable rate (22). One study (11) reported that autistic children learn computer-based gameplay deception tasks faster when they believe they are playing against a computer (non-social condition) rather than a person (social-condition). Another study (23) reported that autistic children who were faster to learn to distrust a deceptive opponent were also faster to learn to deceive during a gameplay paradigm even after controlling for age and VMA (*r* = 0.52).

Other research (28) has reported that autistic children struggle with gameplay deception tasks even when playing against social robots, with no improvement in learning and performance compared with competing against human opponents. One study (16) reported that gameplay deception task performance remained consistent for autistic children with and without the presence of an opponent – though it is unclear whether this was due to social demands or task complexity. However, a recent study (21) indicates that the absence of social context may aid deception performance and learning in autistic adults. During a gameplay deception task against a non-human computer-opponent, autistic adults demonstrated a stronger learning effect for deception than TD adults and were equally likely to engage in successful deception. The researchers speculate that the autistic groups’ successful deception was achieved through observing and learning from opponents’ behaviour and engaging in effortful reasoning about their opponent’s perspective, enhanced by the reduced social demands of the task (21).

One qualitative study (04) suggested that autistic adults decide whether to deceptively omit or reconstruct self-relevant information based upon their reading of social contexts. A second qualitative study (09) proposed that some autistic adults learn from social experience that honesty is not always a useful or desirable quality during interactions with others.

## Discussion

This scoping review summarised the findings of research to date examining deceptive behaviour among autistic children, adolescents and adults. Across the majority of the 28 studies included, a broad pattern of reduced ability and prevalence of deception among autistic children is clear. This raises the question of whether a lack of ability or lack of inclination best explains these findings. While the increased honesty often described as characteristic of autism (e.g. Atherton et al., 2019; Chevallier et al., 2012b; de Schipper et al., 2016) and social motivation theories of autism (e.g. Chevallier et al., 2012a) point to a lack of inclination to deceive, the findings in this review provide compelling evidence for an ability explanation.

Multiple studies showed that autistic individuals often have difficulty understanding the outcomes of deceptive acts (e.g. predicting where an opponent would look for a hidden object). One study reported that non-verbal deception displayed by autistic children is less effective, reflecting developmental diagnostic features which encompass difficulties in non-verbal communication (American Psychiatric Association, 2013). This suggests that although autistic children engage in deception, these attempts may be partially less successful due to inherent issues with maintaining appropriate facial emotion. Furthermore, many autistic children engage in naturalistic deception (suggesting that inclination is intact) though struggle with maintaining their lies (suggesting diminished ability). Applying Talwar and Lee’s (2008) three-stage developmental model, this reflects the *primary stage* of lying (i.e. capable of simple false denials) while TD children of equivalent age would typically have reached the *tertiary stage;* acquiring belief manipulation and verbal lie control. In addition, false denials at the *primary stage* may only represent manipulation of others’ behaviour rather than their beliefs (Jakubowska et al., 2019). Therefore, deception involving reasoning about a target’s beliefs presents a distinct challenge to autistic children below the age of 11 years.

However, deception ability also varies between autistic individuals. In some studies, psychological correlates of deception such as verbal ability, ToM and certain domains of executive function positively related to ability and prevalence of deceptive behaviour. In many of these studies, autistic adolescents and adults’ verbal and mental ages were considerably lower than their chronological ages. Furthermore, of the eight studies involving autistic adults, four were with mixed samples of adults and either children or adolescents (or both). These mixed-sample studies broadly mirrored the findings from research exclusively with autistic children, in which the autistic group were less likely to deceive (and do so successfully) than comparison groups. However, the findings of these four mixed sample studies did not distinguish between younger and older autistic participants. This therefore leaves room for the possibility that deception ability may develop later in life for autistic individuals without co-occurring ID or significantly delayed verbal ability. Indeed, studies with exclusively adult samples of autistic participants present a more complex picture of deceptive behaviour. As one study showed, autistic adults were able to deceive a computerised opponent more frequently and effectively than TD comparisons. It is of note that this participant sample were of above average IQ and were among the oldest (*m =* 33.7 years) of participants across all studies in this review. However, it should be noted that deceiving a target incapable of holding beliefs (i.e. a computer) may not be a true measure of deception – rather, only of mechanisms associated with deception (see Sip et al., 2008). Although it is also clear that autistic adults do at least attempt to engage in naturalistic verbal deception outside of the confines of experimental research. Two qualitative studies described lying by autistic adults in relation to nuanced social reasoning, such as using deception as a form of self-protection and identity management, as well as learning from experience that deception may be of social benefit to others. Verbal ability can develop substantially in autism from childhood into adolescence and adulthood (Bal et al., 2019), as can aspects of ToM and executive function (Cantio et al., 2018). Thus, heterogeneity and fluidity in certain autism characteristics may contribute to increased deception ability for intellectually-able adolescents and adults.

The underlying social and cognitive correlates of deception in autism remain inconclusive. For instance, the relationship between ToM abilities and deception performance in autism has been identified only intermittently by previous studies. Differing task demands cannot fully explain this inconsistency, as some studies report a relationship while other using similar paradigms did not. It is notable that studies tended to use (either formal or informal) measures of false-belief understanding, and no published research to date has employed tests which require participants’ interpretation of a broader range of behaviour, such as the Adult-Theory of Mind (A-ToM) measure (Brewer et al., 2017). However, recent studies suggest that ToM difficulties may be addressed by autistic individuals engaging in effortful reasoning during certain types of deception, utilising working memory and social learning. Indeed, working memory is theorised as central to deception in neurotypical individuals (Sporer, 2016; Walczyk et al., 2014). Compensatory socio-cognitive strategies are used as an alternate route to ToM by some autistic individuals; for example, by consciously connecting past behaviours and context to form interpretations of social behaviour (Livingston et al., 2019). Higher IQ may also aid the learning and application of compensatory strategies for autistic people (Livingston et al., 2020). As such, it may be that autistic individuals (without co-occurring ID) rely more heavily upon executive function processing during deception to enact socially learned strategies as well as effortful reasoning about others’ perspectives. However, no studies have yet specifically measured compensatory strategies in deception, leaving a clear direction for future research.

The extent to which compensatory strategies could be used in more socially demanding instances of deception remains unclear. Studies in the present review demonstrate that recognition of social cues, and the degree to which contexts are perceived as social, remain barriers to deception for autistic children. Social contexts have been shown to have a detrimental effect on the social cognitive functioning of autistic children, while functioning in non-social contexts is less affected (e.g. Chevallier et al., 2014). Although two studies in the current review reported that autistic children can be taught to engage in gameplay and pro-social verbal deception, it is unclear whether this represented improved ability and understanding as opposed to displaying learned behaviours associated with deception. However, the finding that ToM and executive function training increased ability on a gameplay deception task is consistent with other research showing that autistic children and adolescents who receive ToM or executive function training demonstrate improved social outcomes (Kenworthy et al., 2014; Lecheler et al., 2021 but also see Marraffa & Araba, 2016).

### Limitations

The studies included in this review are restricted to those identified through the search terms, databases and reference lists. It is therefore possible that not all relevant research was identified. For example, this review does not contain every published study in which the ‘penny hiding game’ task was used. Multiple studies have used this task as part of a battery of ToM measures. Several of these were screened for the review and excluded as they did not specifically report results from the penny hiding game. Finally, only English language articles were included, potentially limiting the available range of theory, concepts and findings.

### Future directions

This scoping review introduces numerous areas for future research. Deception research thus far has primarily focused on autistic male children and early adolescents, therefore understanding of differences in deceptive behaviour post-childhood (and across genders) is limited. A compensatory framework of deception enables investigation of socio-cognitive strategies, executive function, memory and reasoning, as well as the stage of development at which compensatory strategies emerge. The development of deceptive behaviour has implications for a range of interpersonal behaviour, including reciprocal social skills (Riggio et al., 1987) and impression management (Luo et al., 2017). Considering autistic peoples’ experiences of (and susceptibility to) bullying and exploitation (Cappadocia et al., 2012; Chandler et al., 2019), deception may also be an important self-protective tool. Concerningly, autistic people are disproportionately perceived as deceptive even when truthful (Lim et al., 2021). Therefore, deception research in the context of the criminal justice system has the potential to illuminate further areas of vulnerability.

## Conclusion

The present scoping review has revealed a resurgence of interest in autism and deceptive behaviour over recent years. While many autistic children have difficulties with deception, this review challenges the assumption that autistic people are typically incapable of deceptive behaviour. Prevalence and ability of gameplay and naturalistic deception in autistic individuals varies and are associated with verbal, intellectual and social ability, theory of mind behaviours and domains of executive function, as well as social learning. More sophisticated gameplay and naturalistic deception can be demonstrated by autistic adults without co-occurring intellectual disability. A compensatory lens may help to progress understanding of deceptive behaviour in autism; for example, some autistic individuals appear to use effortful reasoning and learned social strategies during deception to overcome difficulties in taking others’ perspectives. A broader range of qualitative and quantitative methods are required for a nuanced understanding of the development, presentation and utility of this complex social behaviour in autism.

## Supplemental Material

sj-pdf-1-aut-10.1177_13623613211057974 – Supplemental material for Deceptive behaviour in autism: A scoping review Click here for additional data file. Supplemental material, sj-pdf-1-aut-10.1177_13623613211057974 for Deceptive behaviour in autism: A scoping review by Ralph Bagnall, Ailsa Russell, Mark Brosnan and Katie Maras in Autism

## Notes

1. Based on a search on 29 July 2020 in (1) Joanna Briggs Institute (JBI) Evidence Synthesis; (2) Cochrane Database of Systematic Reviews; (3) PubMed; (4) Embase and (5) PsycINFO.
