# Supplementary material for: Deceptive behaviour in autism: A scoping review
Source: Autism. 2021 Nov 26;26(2):293–307. doi: 10.1177/13623613211057974 (PMC8814957; doi:10.1177/13623613211057974)
Supplement: sj-pdf-1-aut-10.1177_13623613211057974 – Supplemental material for Deceptive behaviour in autism: A scoping review [file sj-pdf-1-aut-10.1177_13623613211057974.pdf]

Deceptive behaviour in autism: a scoping review

Supplementary material

Appendix A. Scoping review search strategies
Appendix B. List of included studies (n = 28)
Appendix C. Study characteristics
Appendix D. Study themes and sub-themes
Appendix E. Deception terminology index

Appendix A. *Scoping review search strategies*

Database: **SCOPUS** (03/11/20)

#	Searches	Results
1	lying	103,496
2	deceive*	3,998
3	deceptive	7,763
4	deception	17,312
5	1 OR 2 OR 3 OR 4	127,586
6	autis*	86,405
7	asperger*	6,228
8	“ASD”	37,738
9	6 OR 7 OR 8	100,291
10	5 AND 9	150

(all = title, abstract, keywords)

Database: **APA PsycINFO** (03/11/20)

#	Searches	Results
1	lying	5,777
2	deceive*	1,492
3	deceptive	2,717
4	deception	11,058
5	1 OR 2 OR 3 OR 4	16,897
6	autis*	84,472
7	asperger*	4,850
8	ASD	19,998
9	6 OR 7 OR 8	85,439
10	5 AND 9	153

(all = any fields)

Database: **PubMed** (03/11/20)

#	Searches	Results
1	lying	49,505
2	deceive*	804
3	deceptive	9,007
4	deception	9,007
5	1 OR 2 OR 3 OR 4	52,206
6	autis*	55,852
7	asperger*	2,973
8	“ASD”	25,212
9	6 OR 7 OR 8	63,900
10	5 AND 9	112

(all = any fields)

Appendix B. *List of included studies (n = 28)*

Code	Authors	Country	Aim	Design	Sample	Findings
1	Barbaro et al. (2007)	Australia	To investigate the use of SPDR in children with HFA and AspD, via performance on a task in which they are instructed to act deceptively (e.g., maintaining a neutral expression to disguise their emotion).	Experimental, between groups.	21 male children with HFA (CA = 96.2 months, SD = 19.1)<br>18 male children with AspD (CA = 99.2 months, SD = 18.6)<br>20 male TD children (CA = 80 months, SD = 20.9)	TD children maintained the most effective SPDR (neutralisation of affect) at significantly higher rates of than children with HFA and AspD. TD children used smile suppression (the second most effective SDPR) significantly more than children with HFA, though not AspD children. No differences between HFA and AspD groups on either of these SDPRs. All groups demonstrated equal awareness of the deceptive situation (measured via gaze and tracking behaviours such as looking at box). HFA children showed reduced understanding of SDPR (no difference between TD and AspD children), though group differences disappeared after controlling for VMA.
2	Baron-Cohen (1992)	UK	To examine whether young people with autism are able to engage in a simple game of deception, through replicating Oswald and Ollendick's (1989) findings.	Experimental, between groups.	15 autistic young people (CA = 15.3 years, SD = 2.4)<br>15 young people with ID (CA = 15.44 years, SD = 1.9)<br>15 TD children (CA = 3.8 years, SD = .47).<br>3:1 male-female ratio in the autism group, 1:1 in the TD & ID groups.	Autistic group were equally likely as TD and ID groups to demonstrate object occlusion. Significantly fewer autistic young people demonstrated information occlusion than TD and ID groups. Autistic group were more likely to show the location of the penny before the experimenter guessed, to keep the empty hand open, and failed to hide the hand-penny transfer. Autistic group attempted misinformation significantly less frequently than the ID group (though not the TD group). Author concludes that findings reflect diminished ToM.
3	Bergstrom et al. (2016)	Canada	To examine whether BST could be used to teach children with autism to tell socially appropriate lies.	Non-concurrent multiple baseline intervention design.	3 autistic children. 2 males (5 and 9 years). 1 female (7 years old).	The authors report that within a few sessions of rule teaching, role-play and feedback, all participants told socially appropriate lies in response to gift and appearance sessions. Outcomes were generalised through children being able to respond as taught to novel adults and stimuli. The authors concluded that BST training appears to be an effective approach for teaching socially appropriate lying to autistic children.
4	Davidson & Henderson (2010)	Canada	To review and analyse autistic peoples' autobiographical written accounts of how they choose (and choose not) to disclose their autism.	Qualitative discourse analysis.	45 pre-existing autobiographical narratives by autistic individuals.	Narrative accounts of autistic adults show 'qualified deception' - self-protective identity management for engaging in social contexts - in which autistic adults choose to reconstruct or omit certain details about themselves to disguise their autism. Therefore, authors suggest that autistic individuals can use social strategies for 'keeping safe' which can involve deceptive self-presentation.
5	Fisher & Happé (2005)	UK	To examine whether ToM or EF specific training produce independent outcomes on ToM and EF measures.	Experimental, between groups.	27 children, 20 with diagnosis of autism or ASD. 1 child with AspD. 6 children who met criteria for autism diagnosis based on DSM-IV.<br>ToM condition (CA = 10.5 years, SD = 3.02)<br>EF condition (CA = 10.68 years, SD = 2.68).<br>Control condition (CA = 9.67 years, SD = 1.73)	The ToM training group and the EF training group each improved significantly on PHG performance. The control group did not improve on PHG performance. The authors conclude that the groups trained in ToM and EF generalised their learning to PHG performance.

Code	Authors	Country	Aim	Design	Sample	Findings
6	Gowen et al. (2008)	UK / New Zealand	To examine incongruent movement interference in autism.	Experimental, between groups.	9 autistic adults - HFA (*n* = 2) and AspD (*n* = 7) (3 males, 6 females, CA = 33.9 years, SD = 13.2)<br>12 TD adults (gender not reported, CA = 32 years, SD = 11.8)	8 autistic adults took part in the PHG as part of battery of ToM measures. Autistic adult PHG scores were: 0, 0.33/1, 0.67/1, 0.67/1, 0.671/1, 1/1, 1/1, 1/1. Group mean score for autistic adults not reported but based on the above this would be 0.67/1. TD grouped mean score was reported as 1/1.
7	Hughes et al. (1993)	UK	To examine whether autistic childrens' difficulty with TWT (e.g., Russell et al., 1991; Sodian & Frith, 1992) is primarily due to ToM deficits or an impaired ability to 'disengage' attention from a desired object and to point to where the object is absent.	Experimental, between-groups.	Experiment 1:<br>60 autistic young people (3.6 male : 1 female, CA = 13.3 years)<br>60 young people with ID (3 male : 1 female, CA = 11.5 years)<br>Experiment 2:<br>40 autistic young people (CA = 12.8 years).<br>25 young people with ID (CA = 10.8 years)	More autistic children failed to deceive and indicated the baited box than children with ID. No improvement in 'no opponent' conditions (not requiring ToM) or between verbal or non-verbal deception conditions. Difficulty with object disengagement was replicated in a task with no competitive/deception element (Study 2). The authors conclude that autistic childrens' difficulty with TWT is primarily due to executive (dys)function rather than ToM.
8	Hughes et al. (1997)	UK / France	To examine ToM understanding in relation to social behaviour.	Experimental, between groups.	21 children with PDD: 13 males with autism; 7 males and 1 female with PDD-NOS (CA = 7.4 years, SD = 2)<br>22 TD children: 20 males, 2 females (CA=4.5 years, SD = 1.2)	Little detail provided on PHG performance (as part of battery of ToM measures), but the authors report that the difference between PDD and TD groups was insignificant. The authors note that the PDD group tended towards passing the PHG before false belief tasks, though this effect was not significant.
9	Jaarsma et al. (2012)	Sweden	To discuss truthfulness and lying among autistic people in light of Kantian philosophy and 'ethics of care', including the morality of teaching lie-telling to autistic children and adolescents.	Review/philosophical article.	Excerpts from autobiographical narratives of autistic people.	The authors propose that the strong tendency towards truth in autism will pose difficulties for autistic people, considering the social function(s) played by lying in everyday life. The authors conclude that autistic people should receive training to lie empathically in order to live healthier social lives.
10	Li et al. (2011)	Canada	To examine whether children with autism tell spontaneous verbal white lies and antisocial lies. Also, whether lie-telling in autism is associated with first-order false belief understanding and lie maintenance is associated with second-order false belief understanding.	Experimental, between groups.	19 children with pre-existing clinical diagnoses of Autistic Disorder (*n* = 11), AspD (*n* = 4), ASD (*n* = 2), or PDD-NOS (*n* = 2); (4 female, 15 male, CA = 8.27 years, SD = 1.72)<br>30 TD children (8 female, 22 male, CA = 7.28 years, SD = 0.88)	Autistic and TD children equally likely to tell antisocial and white lies, though autistic children significantly less able to maintain their lies. Antisocial and white lie-telling not associated with false belief understanding in either group. Antisocial lie-telling not associated with second-order false belief understanding in autistic children. Authors conclude lie-telling in autism may rely upon social learning.

Code	Authors	Country	Aim	Design	Sample	Findings
11	Lu et al. (2019)	China	To examine if deception and distrust-based rule learning in autism is impacted by whether participants believed they were playing against a human or non-human / computer opponent (i.e., social vs. non-social context).	Experimental, between-groups.	28 autistic children (5 female, 23 male, CA = 5.83 years, SD = 0.81).<br>28 TD children (5 female, 23 male, CA = 5.53 years, SD = 0.42)	In the social condition, autistic children were less likely to successfully deceive. 84.6% of TD children learned to do this by the end of the task whereas only 23.1% of autistic children did so (with slower learning speeds). In the non-social condition, deception learning performance was closer between groups. Autistic children learned more slowly in the social condition than the non-social condition. Authors conclude that rule-learning for deception tasks in autism is compromised when the context is perceived as social (rather than non-social).
12	Ma et al. (2019)	China	To examine the relationship between EF and deception in children with ASD. The authors hypothesised that autistic children rely upon EF to a greater degree than ToM during self-protective and self-benefiting deception.	Experimental, between groups.	20 autistic children (19 male, 1 female, CA = 11.86 years, SD = 2.27)<br>20 children with ID (19 male, 1 female; CA = 11.73, SD = 2.51)<br>20 TD children (19 male, 1 female, CA = 5.06 years, SD = 0.42)	Autistic children were significantly less likely to tell a self-protective lie than TD children, though not ID children. Fewer autistic children engaged in semantic leakage control than TD children, though not ID children. The majority of autistic children told self-benefiting lies, though fewer than ID and TD children. Working memory, though not ToM or inhibitory control, was found to be significantly positively correlated with lying in the autistic group. The authors suggest that autistic children compensate for impaired ToM through drawing upon working memory when lying.
13	Oswald & Ollendick (1989)	USA	To examine role-taking in autism and its relationship with social interaction skills.	Experimental, between groups.	10 autistic young males (CA = 183 months, SD = 19.4)<br>10 young males with ID (CA = 181 months, SD = 17)<br>The authors note that on the basis of overall cognitive functioning, the autistic group met criteria for intellectual disability.	ID group demonstrated significantly better PHG performance than the autistic group. Across groups, PHG scores were significantly correlated with social interaction skills and the extent to which participants engaged in positive activities with others. The authors suggest that autism may be related to difficulties in managing multiple, simultaneous demands of role-taking and social interaction.
14	Pilowsky et al. (2000)	Israel	To examine and compare ToM ability in schizophrenia, autism and typical development during childhood.	Experimental, between groups.	12 autistic adolescents and adults (11 males, 1 female, CA = 156.33 months, SD = 46.9)<br>12 children with schizophrenia (9 males, 3 females, CA = 146.17 months, SD = 20.17)<br>12 TD children (11 males, 1 female, CA= 102.33 months, SD = 15.92)	Autistic group performed significantly worse on deception task than children with schizophrenia, though not TD children. 83% of autistic children were able to 'trick' the experimenter but only 58% correctly answered the prediction questions. ToM in the schizophrenia group was broadly equivalent in the autism group, though this did not preclude stronger deception performance in the schizophrenia group. The authors suggest that this may reflect specific differences in ability and onset of ToM between groups.

Code	Authors	Country	Aim	Design	Sample	Findings
15	Reinecke et al. (1997)	USA	To test whether autistic young people could be taught to engage in deception during game-play contexts.	Multiple baseline across-subjects intervention design.	3 autistic children/adolescents with co-occurring ID and limited verbal ability. 1 female (12 years) and 2 males (14 years and 9 years).	All 3 participants demonstrated learned deception task skills. Deception task skills were acquired (albeit inconsistently) by 2 of the 3 participants prior to the treatment phase, indicating that the participants had learned these skills pre-intervention. Authors conclude that this demonstrates individuals with autism can learn to deceive even without intensive training.
16	Russell et al. (2003)	UK / Canada	To examine whether TWT is primarily a test of ToM (mentalising/false beliefs understanding) or EF (inhibition, working memory) through comparing performance the AWT when social and deceptive elements of the task are removed.	Experimental, between groups.	Condition 1 (no opponent):<br>20 autistic children (CA = 12.58 years, SD = 2.39)<br>20 children with MLD (CA = 10.33 years, SD = 2.03)<br>Condition 2 (opponent-nondeceptive):<br>20 autistic children (CA = 8.35 years, SD = 1.48)<br>20 children with MLD (CA = 9.40 years, SD = 1.21)<br>Condition 3 (opponent-deceptive):<br>20 autistic children (CA = 8.90 years, SD = 2.92)<br>17 children with MLD (CA = 9.75 years, SD = 1.04).	Autistic children did not perform significantly worse on opponent-deceptive trials than no-opponent trials. Autistic children only scored significantly lower in opponent-non-deceptive condition than children with MLD. No statistical interaction between group (autistic and MLD) and condition (1, 2, 3). The authors suggest that automation removed much of the task challenge across the three conditions for all children (including autistic children). The authors conclude that this may have been in part due to the AWT requiring an inhibition response distinct from the original TWT.
17	Russell et al. (1991)	UK	To examine strategic deception and its relationship with false belief understanding in young children and autistic individuals using a task that requires minimal linguistic and narrative demands.	Experimental, between groups.	11 autistic young people (range = 7.5–17.2 years). Mean age not reported.<br>17 TD children (range = 3– 3.10 years). Mean age not reported.<br>16 TD children (range = 4–4.10 years). Mean age not reported.<br>14 young people with ID (range = 7.2–18.9 years). Mean age not reported.<br>Gender not reported in any groups.	Autistic children were significantly more likely to point to the box containing to the sweet than the empty box on the first trial. This performance was similar to three year old children, whereas four year olds and children with Down's tended to point to the empty box. Similarly, to the three year olds, across the 20 trials, a substantial proportion of the autistic children continued to point to the wrong box (the four year olds and Down's children did not). An association between false belief understanding and task performance was reported. However, the authors suggest that the perseverance of continuing with the wrong box may need further explanation. EF deficits (e.g., inhibition) may prioritise knowledge of physical objects (i.e., reward location) over mental knowledge (i.e., the opponent can be manipulated to choose the wrong box)

Code	Authors	Country	Aim	Design	Sample	Findings
18	San José Cáceres et al. (2014)	UK	To explore performance on the PHG between ID and ID/ASD groups, as a test of non-verbal ToM and as a correlate of everyday social adaptation.	Experimental, between-groups.	56 autistic children with moderate to severe ID (51 male, 5 female, CA = 12.23 years, SD = 3.40)<br>76 children with moderate to severe ID (50 male, 26 female, CA = 12.73 years, SD = 3.32)	ID/ASD group made significantly more errors during the PHG than ID group. ID/ASD group also used significantly fewer tricks than the ID group. VMA was a significant predictor of PHG success in both groups, but ID/ASD children needed higher VMA to succeed. After controlling for VMA, correlations indicated relationships between PHG errors and social insight in the ID/ASD group. Authors suggest that that ID/ASD children may rely more upon verbal skills to account for social difficulties, while ID children use more intact social understanding for social adaptation.
19	Sodian & Frith (1992)	Germany / UK	To explore whether deception difficulties in autism are related primarily to false-belief deficits or if ability to plan and execute competitive, sabotaging behaviours (i.e., not involving belief representation) is also impaired.	Experimental, between groups.	19 autistic young people (8 female, 11 male, range = 6-19 years).<br>29 young people with ID (12 female, 17 male, range = 10-16.7 years).<br>39 TD children (20 female, 19 male, range = 3-5.2 years).	Autistic young people performed poorly on the one box verbal lie deception task compared with TD and ID children and young people. Almost all autistic participants (VMA = 4-5) failed this task, and 60% of autistic participants (VMA = 7-12) passed. The autistic group performed equally well as TD and ID groups on the one box sabotage task. In the two box deceptive pointing task, autistic and ID groups did not differ, and the autistic group performed worse than TD group. Authors reported that false belief understanding was strongest predictor of deception performance in verbal lying and deceptive pointing conditions across groups, above group and VMA. As autistic children succeeded in sabotage when a physical component (i.e., lock) was included, though struggled with no physical component and in deception tasks, the authors conclude that ToM deficits in autism likely explain deception difficulties.
20	Talwar et al. (2012)	Canada	To explore whether children with ASD can spontaneously lie without instruction to do so, and how this relates to ToM.	Experimental, between groups.	26 autistic children - Autistic Disorder (*n* = 19), AspD (*n* = 4), PDD-NOS (*n* = 3) – (22 male, 4 female, CA = 107.19 months, SD = 24.61)<br>27 TD children (22 male, 5 female, CA = 80.4 months, SD = 12.4)	Majority of autistic children spontaneously lied during the task (though fewer than TD group). In comparison to TD children, the autistic group had greater difficulty maintaining their lie during follow up questioning. First order false belief scores were significantly associated with lying across groups, though not within. TD children, who were more likely to lie, also scored more highly on the second order false belief task. Authors suggest that impaired ToM contributes to reduced lying in the autistic group.

Code	Authors	Country	Aim	Design	Sample	Findings
21	van Tiel et al. (2021)	Netherlands / Belgium	To assess the ability of autistic adults to engage in deception in a non-social context.	Experimental, between-groups.	27 autistic adults (13 females, 14 males, CA = 33.7 years, SD = 9.4) 26 TD adults (11 females, 15 males, CA = 29.4 years, SD = 8.5)	Autistic adults were equally likely to deceive a computerised opponent as TD adults. On trials in which deception would produce a higher score, autistic participants were more likely to deceive (and therefore behave optimally) than the TD group. Autistic adults demonstrated a greater learning effect than TD adults, with their performance improving throughout the task. Authors suggest that the autistic group may therefore have utilised social learning ability to account for difficulties with perspective taking.
22	Yang et al. (2017)	China	To examine whether non-social cues help children with ASD to learn to distrust following experiences of deception and also to engage in deception.	Experimental, between groups.	42 autistic children (37 male, 5 female, CA = 5.95 years, SD = 0.84) 38 TD children (27 male, 11 female, CA = 5.73 years, SD = 0.71)	Autistic children were more likely to trust the opponent choose the baited box during social cue trials than TD children. No significant group differences during non-social cue trials between groups. In the deception tasks, autistic children were significantly less likely to point to the empty box than TD children. There was no main effect of condition or group-condition interaction in deception tasks. TD children learned to deceive faster than autistic children in the social condition, but groups performed similarly in the non-social condition. TD children learned quicker in the social than non-social condition, autistic children learned at similar rate in both conditions. No association was found between NVIQ, VIQ or AQ and deception performance in the autism group.
23	Yi et al. (2014)	China / Canada	To examine whether autistic and TD groups would learn to engage in retaliatory deception against an experimenter who had repeatedly attempted to mislead them. Also, to examine whether deception performance correlated with ToM and executive function.	Experimental, between groups.	25 autistic children (22 male, 3 female, CA = 7.57 years, SD = 1.24) 25 TD children (22 male, 3 female, CA = 5.25 years, SD = .64)	Autistic children were less likely than TD children to learn to distrust the experimenter after being repeatedly deceived. Autistic children were also less likely to engage in retaliatory deception than TD children. Autistic children who were faster to learn that they had been deceived by an experimenter were also faster to learn to use deceptive strategies in the task. FB understanding not associated with trust or deception task in either group. Autistic children with higher EF scores learned to deceive more quickly. However, after controlling for CA and VMA, no correlation between EF and deception performance in the autistic group (as the EF test was highly correlated with VMA).

Code	Authors	Country	Aim	Design	Sample	Findings
24	Yirmiya et al. (1996)	Israel	To examine deception ability and understanding in autism using a simpler paradigm than used by previous researchers and in a participant sample with an older mental age than previous studies. To examine the ability to disengage from salient knowledge (e.g., object location) and salient physical objects (i.e., false trails) during deception.	Experimental, between-groups.	14 autistic individuals (10 male, 4 female, CA = 21.3 years, SD = 5.9)<br>15 individuals with ID (13 male, 2 female, CA = 20.1 years, SD = 6.2)<br>16 TD children (12 male, 4 female, CA = 8 years, SD = 1.4)	Autistic group were less likely to engage in deception than the TD group. Autistic participants were less able than the TD group to correctly predict the consequences of their deception. Of the autistic participants who laid a false trail of footprints, fewer also understood belief manipulation compared with the TD group (though not ID). The MAs and CAs of participants in the autism group who successfully erased or laid false trails did not differ the participants who were unable to do so. Authors suggest the findings may indicate ToM deficits associated with autism impair deception understanding to a greater extent than ability to disengage from a salient object.
25	Yirmiya et al. (1996)	Israel	To examine the specificities of ToM deficits in autism through comparing ToM ability in autism, ID and TD groups across tasks of varying complexity (including deception).	Experimental, between groups.	25 autistic adolescents and adults (CA =20.5 years, SD = 6.2)<br>21 adolescents and adults with ID of unknown origin (CA = 21.11 years, SD = 6.5)<br>19 adolescents and adults with Down syndrome (CA = 23.11 years, SD = 6.8)<br>21 TD children (CA = 7.9 years, SD = 1.6)	When matched on performance and VMA the TD group performed better than all clinical groups (including autistic) in using deception and predicting its outcome. No significant differences between the autistic and ID groups. VMA was a positive predictor of deception performance in the autistic group. Authors note that their finding that the ID groups did not outperform the autism group on deception measures differs from previous research - suggest previous research included participants with less significant ID than participants in the current study.
26	Yokota & Tanaka (2013)	Japan	To examine whether autistic and TD children differ in age when they begin to demonstrate 1) concealing (hiding information from others) and 2) strategic (hiding and actively providing wrong information) deception.	Experimental, between groups.	11 autistic children – younger group (10 male, 1 female, CA = 108.8 months, SD = 51.5)<br>18 autistic children – older group (14 male, 4 female, CA = 128.4 months, SD = 28.8)<br>21 TD children – younger group (13 male, 8 female, CA = 49.6 months, SD = 4.1)<br>26 TD children – older group (13 male, 13 female, CA = 70 months, SD = 5.8)	Gameplay paradigm - VMA predicted concealing of information in autistic children, though not strategic deception. Older autistic children performed better than younger autistic children at concealing (though not strategic deception). The difference in concealing between autistic and TD children with VMA of 3-6 years was of marginal significance. The difference between autistic and TD children (excluding those with VMA of 3-6 years) in strategic deception was non-significant.<br>TRP – Of the 13 autistic children who peeked at the cards, 11 concealed this information by lying, compared with 19 TD children (of 22 who peeked) who lied. Of the 11 autistic children who lied, 7 also strategically deceived (with no association with participant age). No significant difference between TD and autistic children in strategic deception frequency.

Code	Authors	Country	Aim	Design	Sample	Findings
27	Yokota & Tanaka (2020)	Japan	To examine whether executive function is associated with capacity (accuracy score) and fluency (reaction time) of deception in autism.	Experimental, between-groups.	21 autistic children (gender not reported, CA = 114.24 months, SD = 17.68)<br>29 TD children (gender not reported, CA = 199.7 months, SD = 20.19)	No significant difference in deception accuracy between groups, though autistic group had significantly slower RT (*d* = .71). Age and EF planning were significantly negatively correlated with RT in the autistic group, though only age > RT in the TD group. No other significant relationships between variables in either group.
28	Zhang et al. (2019)	China / Canada / Sweden	To examine whether children with autism could learn to distrust and deceive a social robot and the extent to which deception learning was associated with childrens' perception of the robot (e.g., perception of human-like qualities). And whether this differed from distrust and deception learning against a human opponent.	Experimental, between groups.	Robot condition:<br>- 20 autistic children (18 males, 2 females, CA = 6.79 years, SD = 0.93)<br>- 20 TD children (17 males, 3 females, CA = 6.35 years, SD = 0.56)<br>Human condition (dataset from Yang et al. (2017), with additional recruitment for TD group):<br>- 17 autistic children (14 males, 3 females, CA years = 6.17, SD = 0.77)<br>- 22 TD children (20 males, 2 females; CA = 6.31 years, SD = 0.65)	Autistic children were less likely to distrust deceive the social robot and slower to learn to do so than TD children. Deception performance was not associated with anthropomorphic thinking in either group. Authors compared findings with social robot against previous study with human target (Yang et al., 2017). Autistic children were more likely to distrust and learn to distrust the robot than the person (no difference for TD group between conditions). Neither autistic or TD children were no more likely to learn to deceive or engage in deception in either condition. Authors conclude that autistic children demonstrated some social learning of deception against a social robot, though this this was far less efficient than TD children.

Note: Asperger's Disorder (AspD), Autism Spectrum Disorder (ASD), Automated Windows Task (AWT), Behavioural Skills Training (BST), Chronological age – mean (CA), Executive Function (EF), High Functioning Autism (HFA), Intellectual Disability (ID), Mental Age (MA), Moderate Learning Disability (MLD), Penny Hiding Game (PHG), Pervasive Developmental Disorders (PDD), Pervasive Developmental Disorder-Not Otherwise Specified (PDD-NOS), Reaction Time (RT), Self-Presentation Display Rules (SPDRs), Standard deviation (SD), Temptation Resistance Paradigm (TRP), The Windows Task (TWT), Theory of Mind (ToM), Typically developing (TD), Verbal Mental Age (VMA)

Appendix C. *Study characteristics*

Paper characteristics	N	Paper reference code
Year		
1989-2000	10	02, 07, 08, 13, 14, 15, 17, 19, 24, 25
2001-2010	5	01, 04, 05, 06, 16
2011-2021	13	03, 09, 10, 11, 12, 18, 20, 21, 22, 23, 26, 27, 28
Location		
UK	8	02, 05, 06, 07, 16, 17, 18, 19
China	5	11, 12, 22, 23, 28
Canada	4	03, 04, 10, 20
Rest of the world	11	01, 08, 09, 13, 14, 15, 21, 24, 25, 26, 27
Methods		
Quantitative	26	01, 02, 03, 05, 06, 07, 08, 10, 11, 12, 13, 14, 15, 16, 17, 18, 19, 20, 21, 22, 23, 24, 25, 26, 27, 28
Qualitative	2	04, 09
Deception measure		
Hide and seek	10	11, 12, 14, 22, 23, 24, 25, 26, 27, 28
Penny hiding game	7	02, 05, 06, 08, 13, 15, 18
Temptation resistance paradigm	4	10, 12, 20, 26
The Windows Task	3	07, 16, 17
Autobiographical narratives	2	04, 09
Undesirable gift paradigm	1	10
Other	4	01, 03, 19, 21
Mean age (autistic group)		
5-8 years old	9	01, 03, 08, 10, 11, 20, 22, 23, 28
9-11 years old	6	05, 12, 15, 16, 26, 27
12-18 years old	7	02, 07, 13, 14, 17, 18, 19
19-29 years old	2	24, 25
30-34 years old	2	06, 21
Unknown adult age	2	04, 09
% of autistic male participants		
34-58%	3	06, 19, 21
59-80%	6	02, 03, 07, 10, 15, 24
81-100%	13	01, 08, 11, 12, 13, 14, 16, 18, 20, 22, 23, 26, 28
Gender not reported	6	04, 05, 09, 17, 25, 27

Appendix D. *Study themes and sub-themes*

Themes*	N	Paper reference code
Deception ability and prevalence		
Gameplay deception	22	02, 05, 06, 07, 08, 11, 12, 13, 14, 15, 16, 17, 18, 19, 21, 22, 23, 24, 25, 26, 27, 28
Naturalistic deception	7	01, 04, 09, 10, 12, 20, 26
Psychological correlates of deception		
Verbal, intellectual and social ability	14	01, 02, 10, 13, 14, 17, 18, 19, 22, 23, 24, 25, 26, 27
Theory of Mind abilities	14	02, 05, 07, 10, 12, 14, 16, 17, 19, 20, 21, 23, 24, 25
Executive Function	8	05, 07, 12, 16, 19, 23, 24, 27
Social learning		
Training	3	03, 05, 15
Social contexts	8	04, 09, 11, 16, 21, 22, 23, 28

* *Note* - Some studies cover multiple themes

Appendix E. *Deception terminology index*

Review classification	Study terms	Used in paper codes
Gameplay deception	Deception	02, 05, 08, 11, 14, 15, 16, 19, 22, 24, 25, 27, 28
	Strategic deception	07, 17, 21, 26
	Retaliatory deception	23
	Concealing deception	26
	Naturalistic deception*	18
	Self-benefiting lies	12
	Role-taking	13
Naturalistic deception		
Self-protective deception	False denials	20
	Antisocial lies	10
	Strategic deception	26
	Concealing deception	26
	Qualified deception	04
	Self-protective lies	12
Pro-social deception	White lies	10
	Socially appropriate lies	03
Non-verbal deception	Self-presentation display rules	01
	White lie ability	10

* *Note* – this study defined the penny hiding task as a naturalistic game
